# Supplementary material for: Intrathecal Synthesis Index of Specific Anti-Treponema IgG: a New Tool for the Diagnosis of Neurosyphilis
Source: Microbiol Spectr. 2022 Feb 9;10(1):e01477-21. doi: 10.1128/spectrum.01477-21 (PMC8826818; doi:10.1128/spectrum.01477-21)
Supplement: SUPPLEMENTAL FILE 1 — Supplemental material. Download SPECTRUM01477-21_Supp_1_seq4.pdf, PDF file, 0.2 MB [file spectrum01477-21_supp_1_seq4.pdf]

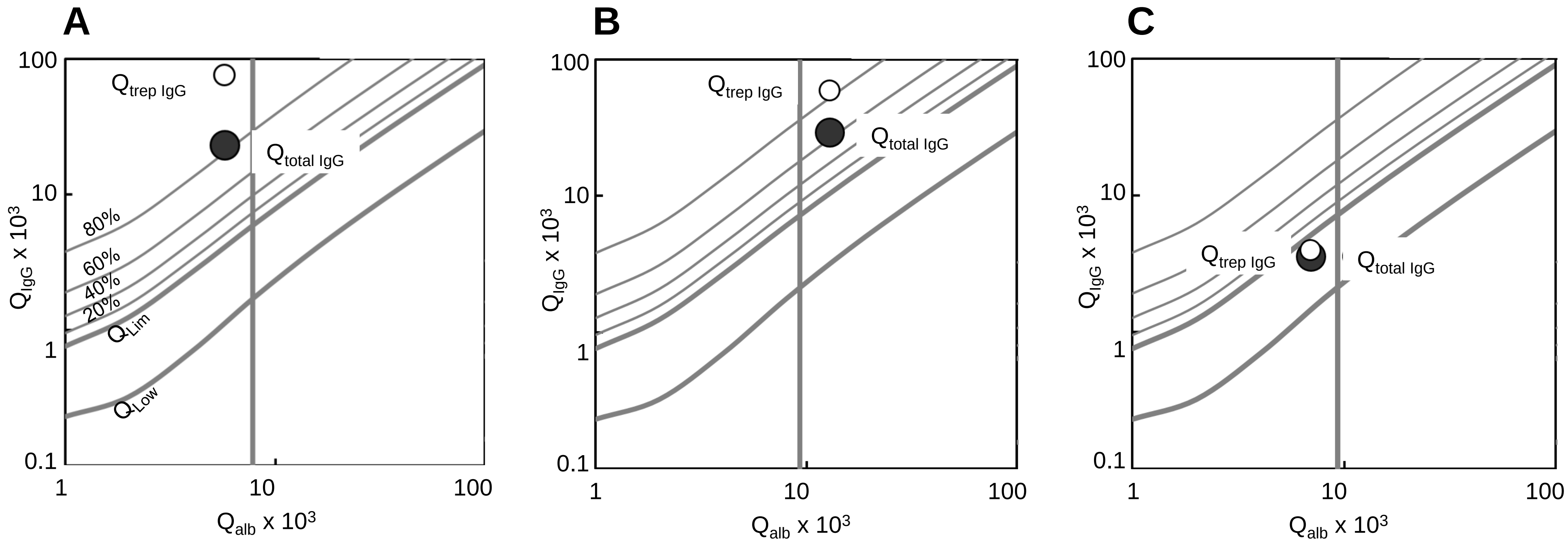

Supplementary figure1. Reiber diagram showing $Q_{trep\ igG}$ (white circle) and $Q_{total\ IgG}$ (black circle) from NS1 (A, B) and NS2 (C) patients with (B) or without Blood Brain Barrier disruption (A,C).

The thick hyperbolic lines represent QLow(IgG) and QLim(IgG) calculated according to $Q_{Lim}IgG = 0.93x\sqrt{(Qalb^2+6x10^{-6})} - 1.7\ x10^{-3}$ and thin hyperbolic lines represent IgG fraction produced in CSF. The vertical line represents the **limits** of the albumin-quotient calculated for age >4 years according to $Q_{Lim}Alb=(4+age/15)x10^{-3}$
